# Social employee well-being challenges of academics in the hybrid work environment

**DOI:** 10.3389/fpsyg.2025.1524804

**Published:** 2025-02-07

**Authors:** Rudo Rachel Marozva, Anna-Marie Pelser

**Affiliations:** ^1^North-West University Business School, North-West University, Mahikeng, South Africa; ^2^Global, Innovative, Forefront, Talent Management (GIFT), Mahikeng, South Africa

**Keywords:** hybrid work environment, sense of belonging, social connections, social employee well-being, work relationships

## Abstract

The complex nature of the hybrid work environment impacts on the overall well-being of employees. There is limited research on how the hybrid work environment has impacted the social dimension of employee well-being, particularly among academics in higher education institutions. The aim of the study was to explore and understand social well-being challenges that academics face as a result of the hybrid work environment. The study adopted a cross-sectional qualitative research approach and used semi-structured interviews using an interview guide to collect data. The sample included 23 academics who are representatives of the three campuses of North-West University. Thematic analysis was used to analyse the data. Three themes emerged from the analyses of data: social connections, sense of belonging and work relationships. The social connections theme had four sub-themes: social isolation, informal communication breakdown, limited networking and personality conflict. Sense of belonging theme entailed four sub-themes: lack of resources, work-life imbalance, onboarding challenges and non-inclusive work environment. Relationships theme had three sub-themes: lack of trust, reduced social support and delayed feedback. Experiences of the research participants are noted to comprehend the negative impact of the hybrid work environment on social employee well-being. The findings suggest that social well-being challenges that academics face are heightened by the nature of the industry, personality traits and the context of the study which is Africa. This requires HEIs to explore the internal and external environment when managing social well-being challenges that academics face as a result of the hybrid work environment.

## Introduction

Several changes have taken place in higher education institutions (HEIs) in the past decades and most of these changes impact employee well-being of academics (Heiden et al., 2021). One of the changes that have been experienced by HEIs in the past decade is the advent of the hybrid work environment (HWE) to mitigate the negative effects of the COVID-19 pandemic. Iwu et al. (2022) argue that HEIs across the globe embraced remote working arrangements that were not planned. Conversely, Wörtler et al. (2021) note that the HWE was introduced in HEIs prior to the pandemic as a retention strategy. Gutman et al. (2024) conceptualize HWE as a flexible work arrangement which allow employees to spend some of their working time in the office and sometime remotely, usually at home. According to Budiman et al. (2022), the HWE can lead to work-life imbalance and increased workload on employees. Babapour Chafi et al. (2022) highlight that the HWE result in loss of human connection which leads to both professional and social isolation. Such isolation leads to anxiety and stress and impact employee well-being. Angeline (2022) contend that stress and anxiety which negatively impact psychological and mental well-being compromises social employee well-being.

Four theories underpinned the study, the job demands-resources theory developed by Bakker and Demerouti and was first introduced in 2001, the job demands control support theory developed by Karesk and Theorell in 1989 and the need to belong theory by Baumeister and Leary developed in 1995. JD-R model categorises job characteristics into job demands and job resources (Demerouti et al., 2001). Job characteristics like workload, conflict, and complex tasks fall under the category of job demands (Bakker and Demerouti, 2018). Job resources entail characteristics of the job like social support, opportunities to be promoted and the freedom to choose work location and time (Tummers and Bakker, 2021). The JDCS theory was initially developed by Karesk in 1976 and later developed by Karesk and Theorell in 1989 to include social support (Theorell, 2013). According to Dutheil et al. (2020), the theory focuses on contradicting tasks in the work environment and divides them into job demands, job control and social support. Job control entails an individual’s belief in effecting change in their work environment, including autonomy and discretion on how tasks are completed (Fila, 2016). Job demands entail the physical, social, and organisational aspects of the job that require physical or mental effort; overall workload encompasses job demands (Zou et al., 2024). The need-to-belong theory suggests that the need to belong is a fundamental human need and the desire to maintain lasting and important interpersonal relationships is crucial among human beings (Baumeister and Leary, 1995).

## Social employee well-being

Pagán-Castaño et al. (2020) highlight that social well-being at the workplace deals with relationships and the quality of interactions and relations at all levels in the organisation are central to social employee well-being. Hennicks et al. (2022) indicate that social well-being entails the extent to which individuals function well in their social lives, and work relationships and how they can work well with colleagues. The writers contend that workplace social well-being is an integral component of well-being at the workplace. According to Sakka and Ahammad (2020), effective relationships at the workplace and effective internal communication enhance social well-being at the workplace and are dependent upon interaction among employees.

Colenberg et al. (2021) highlight that most of the conceptualisation of workplace social well-being relies on the work of Keyes (1998). The five dimensions as proposed by Keyes were also adopted by Redelinghuys and Rothmann (2020) to comprehend what entails social well-being at work. According to Redelinghuys and Rothmann (2020), acceptance looks at accepting the diversity of colleagues, actualisation entails belief in the potential of the organisation, team, and colleagues’ potential while coherence looks at the meaningfulness of organisational and social relations. The writers note that contribution looks at the value individual work adds to the team, department, and organisation while integration is a sense of communal connectedness and belonging to the organisation as a community.

Social well-being at work is considered as a feeling of embeddedness in meaningful communities, having short-term interactions or connections and long-term relationships with others (Fisher, 2014 in Colenberg et al., 2021). The feeling of embeddedness relates to the need to belong, whereas social connections include noticing other people’s presence and the deliberate exchange of information; it entails seeing, hearing, smelling as well as touching other people. Long-term relationships involve having positive work relationships both vertically and horizontally at the workplace (Pipera and Fragouli, 2021). According to Vanderbilt University (2021), social interactions are important as they positively impact other employee well-being dimensions such as psychological and mental dimensions. Long-term relationships build resilience among employees, helping them to manage stressful conditions (Vanderbilt University, 2021). Interactions and relationships are crucial for health and well-being and this need is also evident in the workplace where employee connection is important (Colenberg et al., 2021).

While Redelinghuys and Rothmann (2020) conceptualize social well-being at work as having five dimensions, Colenberg et al. (2021) discuss social well-being at the workplace as having three dimensions – belonging, social interactions or connections and relationships – and it is based on the conceptualisation of workplace social well-being by Fisher (2014). The writers contend that the dimensions proposed by Keyes mirror the conditions that contribute to social well-being and do not explain what social well-being is. It is also important to note that the five dimensions discussed by Redelinghuys and Rothmann (2020) can be grouped into the three dimensions that were highlighted by Colenberg et al. (2021). Actualisation overlaps with the feeling of belonging as well as relationships; contribution can be found in all three dimensions; and integration falls under the feeling of belonging, acceptance under social interaction and coherence under long relationships. This study adopted social well-being at the workplace as compromising three dimensions – belonging, social interactions, or connections and relationships.

## Employee social well-being of academics

Based on the discussion of social employee well-being above. The social well-being of academics entails academics’ sense of belonging, work relationships and social connections. Pre-pandemic, the well-being of academics was in a crisis as one in four educators considered leaving the industry due to stress associated with the industry and the crisis has been on the rise because of the pandemic (Jayman et al., 2022). Shen and Slater (2021) argue that the academic profession is characterized by high levels of stress which contribute to compromised well-being of academics. Role ambiguity among academics negatively impacts their well-being. Wei and Ye (2022) note that excessive workload results from role ambiguity where academics are teachers, tutors and counselors and high pressure at work impacts work-life balance, which impacts well-being. This indicates that the job quality of academics is not great. According to Fana et al. (2020), job quality negatively impacts the social well-being of employees due to its impact on social and professional isolation. Social isolation has been found to impact the sense of belonging, work relationships and social connections. Therefore, considering the job quality of academics one can conclude that their social well-being is compromised.

According to Wray and Kinman (2021), changes in teaching and student support during the COVID-19 era placed more demands on the profession which is already exposed to deteriorating well-being. Mayya et al. (2021) posit that a lack of work-life balance in HEIs compromises the well-being of academics. All these expose academics to burnout and stress. The stress and burnout associated with being an academic impact negatively on the psychological well-being of academics. According to Angeline (2022), compromised mood and mental challenges impact negatively on meaningful work relationships. Thereby, one can infer that the stress and burnout that academics endure impact their social well-being as well.

The well-being of academics in developing countries is also compromised by poor infrastructure and accessibility which are critical in a HWE (Rathnayake et al., 2022). Jayman et al. (2022) indicate that internal stressors among academic employees include taxing workloads and lack of autonomy which have been exacerbated by the COVID-19 pandemic including lack of resources. Naidoo-Chetty and Du Plessis (2021) note that insufficient resources impacted negatively on the well-being of academics. Liu et al. (2020) argue that the provision of work resources enhances a sense of belonging among employees via instrumental support. Similarly, Rasool et al. (2021) link resource provision with improved social well-being. The authors contend that instrumental support which is enhanced by resource provision enhances perceived organisational support which in turn enhances a sense of belonging. One can also suggest that the social well-being of academics is compromised by lack of resources which are critical in enhancing a sense of belonging.

Work support through supervisors or co-workers enhances job satisfaction and job satisfaction is an antecedent of employee well-being (Malik and Allam, 2021). Ahmad et al. (2020) highlight that co-worker support is salient among academics in HEIs. While work support is considered important among academics, co-worker support in the higher education industry is negatively affected by toxic leaders. Mahlangu (2020) notes that toxic leaders who do not care about the well-being of employees create toxic relationships among peers. Mayya et al. (2021) indicate that work colleagues, who were supposed to be supportive, were a source of stress among academics in HEIs. Singh (2024) also concurs that work relationships among academics are compromised by toxic leaders. The author notes that academics are exposed to intimidating, discriminating and demoralising mentors. Baloyi (2020) highlights that these toxic leaders destroy human relationships. Therefore, based on the type of leadership characterising HEIs academics’ work relationships are not meaningful, compromising their social employee well-being.

In conclusion, the social well-being of academics is compromised by toxic leaders found in HEIs; they compromise work relationships and the sense of belonging of academics. The job quality of academics also impacts negatively on the social well-being of academics. Work overload impacts on sense of belonging, social connections and work relationships of academics as it leaves academics without time to connect with others and cultivate positive work relationships. Lack of resources which is common in HEIs, impacts the social well-being of academics as it is directly linked with perceived organisational support, which in turn enhances a sense of belonging.

## Rationale of the study

An analysis of literature on academics workplace well-being indicates that most of the studies have dealt with psychological employee well-being. While these studies have contributed much on academics workplace well-being, the social employee well-being dimension has not been well-researched especially in the South African context (Hennicks et al., 2022). The main aim of the study was to explore social employee well-being challenges of academics in HEIs due to the HWE using qualitative methods. The qualitative method enabled the researchers to understand the subjective experiences of academics in the HWE.

An in-depth understanding of academics social well-being challenges will help HEIs to understand the job demands that academics experience due to the HWE and provide required resources to mitigate the negative effects of the job demands in the HWE. Understanding the social well-being challenges resulting from the HWE will enable HEIs to have a holistic approach in managing academics employee well-being as social employee well-being is an important dimension of overall employee well-being (Rahman et al., 2020). Oversight of social employee well-being may negatively impact on academics’ sense of belonging, work relationships and social connectedness among academics in the HWE. The assumption is that most of the drawbacks of the HWE impact negatively on academics’ sense of belonging, work relationships and social connections. The study’s primary objective is to comprehend social well-being challenges that academics face as a result of the hybrid work environment. Therefore, the current study is guided by the following research question: What social well-being challenges are academics facing or have faced in the HWE?

## Materials and methods

Participants of the study were selected from a public higher education institution in the North-West Province, South Africa. North-West University is a public higher education institution located in the North-West Province of South Africa. The University was established in 2004 after a merger between Potchefstroom University of Christian Higher Education and North-West University formerly known as the University of Bophuthatswana. It has three campuses in Potchefstroom, Mahikeng and Vanderbijlpark.

University communication channels were used to advertise the study and recruit research participants. Purposive sampling was used to select research participants, it allowed the researchers to select specially qualified research participants to collect the required data. Adopting purposive sampling for the study enabled the researchers to select research participants that helped to answer research questions and satisfy the objectives of the study. According to Obilor (2023), purposive sampling is mostly used in studies where the researcher wants to gain detailed knowledge about a specific phenomenon.

23 semi-structured interviews were conducted. The sample size of 23 was sufficient for qualitative analysis. Moser and Korstjens (2018) indicate that saturation could be reached with 10 participants in a phenomenological study. According to Guest et al. (2020), six to seven interviews can capture the majority of themes in qualitative research. 65% of the research participants were females while 35% were males. 43% work from Vanderbijlpark campus while 35 and 24% were based at Potchefstroom campus and Mahikeng campus, respectively. 52% of the research participants were lecturers, 35% were senior lecturers, 4% associate professors and 9% were professors. 52% of the research participants followed a flexible hybrid working model, 31% were on a semi-flexible model while 17% used a fixed hybrid working model. 61% of the research participants were millennials, 35% generation X and 4% were baby boomers.

Phenomenology, particularly interpretive phenomenology, was adopted. The study sought to understand the social well-being challenges of academics who work in the HWE, and phenomenology enabled the researchers to understand these from the perspective of research participants. Adopting phenomenology helped the researchers to understand the research subject, which is social well-being challenges at the workplace and the HWE, rather than to predict outcomes on the subject. Tomaszewski et al. (2020) highlight that the result of phenomenology is to deeply understand a research subject rather than to predict outcomes.

### Data collection

To understand and elaborate on the social well-being challenges that academics experience in the HWE, online semi-structured interviews were conducted using an interview guide. The guide was made up of two sections. In Section A research participants were asked demographic questions. Demographic questions helped the researchers to describe the research participants and to do an in-depth analysis of the findings. Section B comprised of open-ended study-specific questions that helped the researchers to answer research questions. The adopted data collection method allowed the researchers to solicit for rich responses from the participants as the method allows the use of probing questions to gain additional information and reflections (Osborne and Grant-Smith, 2021). Questions that participants were asked included: How has the HWE affected your sense of belonging to the organisation? which challenges do academics face in the HWE which compromise work relationships. In what ways has the HWE compromised social connectedness among academics in the HWE?

### Data analysis

Thematic analysis was used to understand social employee well-being challenges that academics face in the HWE. Kiger and Varpio (2020) explain that thematic analysis is a flexible method that seeks to understand experiences, thoughts, or behaviors. It goes beyond describing data, it also interprets the data (Islam and Aldaihani, 2022). The aim of the study supported the adoption of thematic analysis. Kiger and Varpio (2020) argue that thematic analysis is the best method in the case where the researcher seeks to explore lived experiences of research participants.

The Braun and Clarke (2006) six-phase process for thematic analysis was adopted. The first stage entailed being acquainted with the data and identifying items of interest while stage two entailed initial generation of emerging codes and stage three involved distinguishing patterns to form themes (Neuendorf, 2018). The fourth stage required the researchers to evaluate if emerging themes answer research questions, the last two phases entailed analysing each theme and putting together the narrative and data segments, respectively.

Manual coding was used to analyse the data. Coding relates to identifying portions of meaning in data and labeling the meaning with a code (Linneberg and Korsgaard, 2019). An inductive approach to coding was adopted which entail using phrases by the research participants. Linneberg and Korsgaard (2019) suggest that inductive coding is ideal for an exploratory study. Short phrases and words were generated from data taking care not to destroy the essential properties of the data. Similar codes were clustered together to form categories which were further refined to form sub-themes and themes.

### Trustworthiness

Threats to credibility were reduced by respondent validation which entails returning data transcriptions to participants to verify the accuracy of the statements captured (Dangal and Joshi, 2020). The researchers provided an audit trail by detailing how data were collected, how themes in the analysis were derived and how decisions were made throughout the study to increase dependability and confirmability (Carcary, 2020). Finally, the researchers used good thick descriptions to ensure transferability. Creswell and Creswell (2018) indicate that this is achieved by describing the situation with enough detail, allowing readers to conclude on the data presented. Writing down notes during interviews and memoing as soon as possible after interviews are some of the practices that the researchers employed to deal with reflexivity.

### Results

The aim of the research was to understand social employee well-being challenges that academics face in the hybrid work environment. Three themes emerged from the data: social connections, sense of belonging and relationships. The table below summaries the themes and sub-themes of the study (Table 1).

**Table 1:** Summary of themes and sub-themes.

Theme	Sub-theme
1. Social connections 2. Sense of belonging 3. Relationships	1.1 Social isolation 1.2 Reduced informal communication 1.3 Limited networking 1.4 Personality conflict 2.1 Lack of resources 2.2 Work-life imbalance 2.3 Onboarding challenges 2.4 Non-inclusive work environment 3.1 Lack of trust 3.2 Reduced social support 3.3 Delayed feedback

## Social connections

Social connections relate to experiences of being connected and close to others (Haim-Litevsky et al., 2023). Vilnai-Yavetz and Rafaeli (2021), indicate that social connections or interactions are psychological tools that enhance employee well-being, and physical spaces are affordances of social connection at the workplace. The HWE has compromised social connections at the workplace. Charpignon et al. (2023) note that misaligned work schedules jeopardize opportunities for work colleagues to interact. Social connection challenges result from reduced social interactions in the HWE (Hvidt-Andersén et al., 2024). The social connections or interactions theme was the most mentioned theme. The theme had four sub-themes: social isolation, informal communication breakdown, limited networking, and personality conflict.

### Social isolation

Most of the participants noted that while the HWE is a good working environment it has reduced physical interaction among work colleagues. Participants indicated that in most cases it is unlikely to be in the office at the same time as your colleagues as most of the participants highlighted that they followed a flexible hybrid model and report to the office when they consider it highly necessary.

*“The face-to-face physical interaction is missing …aaaaah it will always be missing”* (RP1, female, 52 years, lecturer, Potchefstroom campus).

*“Some things are better said and engaged in person…. Hybrid has killed off the physical phenomenon”* (RP3, male, 29 years, lecturer, Mahikeng campus).

*“…you do not get to see people as much as you would love to because sometimes you can go a week or two without seeing your colleagues”* (RP15, male, 52 years, senior lecturer, Mahikeng campus).

*“… that sense of community is slowly disappearing with the hybrid work”* (RP21, female, 34 years, lecturer, Potchefstroom campus).

Babapour Chafi et al. (2022) note that a systematic review of the literature on the HWE indicated that social isolation was one of its detrimental effects. Correspondingly, Becker et al. (2022) believe the HWE results in extensive feelings of loneliness among workers when employees are working away from the office. Lindeberg et al. (2023) contend that social isolation impacts the well-being of employees. Findings are in line with the need to belong theory that emphasize the importance of frequent interactions in well-being.

### Informal communication breakdown

The findings of the study indicate that informal communications that academics have when they meet their colleagues in the corridors are important in enhancing social connections at the workplace. However, some academics highlighted that informal communications have been reduced by the HWE. One participant noted that at times when they report for work at the office, they will be the only ones there. Another participant noted that before the HWE, academics could pop into each other’s office for informal talks, and this is not possible anymore with the HWE.

“*There are times that I am the only one in my corridor working on campus…. there will be no one to talk to”* (RP10, female, 39 years, lecturer, Vanderbijlpark campus).

*“It is now impossible to just talk to your colleague and find out how they are doing before engaging on other issues”* (RP8, male, 47 years, lecturer, Vanderbijlpark, Campus).

Informal communication which depends on physical proximity has been affected by the HWE (Begemann et al., 2024). According to Koch and Denner (2022), informal communication lubricates the social wheels of an organisation and is a mechanism where employees find opportunities to interact with their colleagues. Informal talks are critical in creating social connections in the workplace and the HWE has reduced these (Hvidt-Andersén et al., 2024). Diab-Bahman and Al-Enzi (2020) contend that autonomy in terms of work location changes the occurrence and nature of communication and decreases face-to-face informal communication. Oppong Peprah (2024) notes that while informal communication is important in enhancing connections, it has been negatively impacted by the HWE. It has affected the social bonding of employees which increases isolation and loneliness among employees (Begemann et al., 2024).

### Limited networking

Networking among academics is very crucial. Academics find opportunities to network at workshops and conferences as well as meetings. A lot of academics noted that even conferences where academics found opportunities to network are now virtual with limited interface which harms possibilities to network and enhance social interactions. Some participants felt the HWE has reduced networking opportunities for academics.

*“Hybrid has reduced networking opportunities… conferences are now hybrid, and networking virtually is difficult”* (RP7, female, 41 years, lecturer, Vanderbijlpark campus).

*“With training and workshops taking place virtually likelihood of meeting colleagues and networking has been reduced”* (RP4, female, 37 years, Vanderbijlpark, Campus).

*“Though we still have face-to-face training and workshops, 90% of these are now done virtually”* (RP21, female, 34 years, lecturer, Potchefstroom campus).

Babapour Chafi et al. (2022) note that employees’ networks tend to be static and soiled due to working virtually. Similarly, Ahmad et al. (2020) indicate that the HWE has impacted collaborations among employees. The HWE has the potential to exclude underrepresented talent from important networks that are critical in career growth (McRae and Kropp, 2022). Correspondingly, Sokolic (2022) argues that opportunities to build connections and networks are reduced among employees because of the HWE. The findings of the study are in line with Hughes and Davis (2021) study on interns working in the HWE. The study linked reduced networking opportunities to the HWE.

### Personality conflict

Two participants noted that they are extroverts and work well when they physically interact with their colleagues. They felt as extroverts the HWE conflicted with their personalities as it enhanced a sense of loneliness.

*“Generally, I love interacting with people, the HWE makes it difficult for people to interact more often”* (RP2, female, 55 years, lecturer, Vanderbijlpark campus).

*“I love to be everywhere, I am a very curious person, I love meeting different people… this is not so with hybrid work… some trainings are virtual only”* (RP10, female, 39 years, lecturer, Vanderbijlpark campus).

According to Drayton (2024), individuals who are extroverts get much of their energy around people and they tend to miss their work colleagues more in the HWE. On the other hand, the findings of the study also indicated that demographic differences also had an impact on the challenges that academics felt because of the HWE. Academics between the ages of 31 and 40 who are mostly millennials felt the HWE created a haven for individuality, which they appreciated.

## Sense of belonging

Virtual connections which are common in the HWE diminish employees’ sense of belonging to the organisation (De Smet et al., 2022). Similarly, Drayton (2024) contends that the need to belong which enhances a sense of belonging when fulfilled is thwarted by the physical distances in the HWE. According to Hincapie and Costa (2024), the complexity of the HWE makes the sense of belonging to the organisation to be a challenge. The sense of belonging theme produced four sub-themes. The sub-themes include a lack of resources, work-life imbalance, onboarding challenges and a lack of an inclusive work environment.

### Lack of resources

Some participants noted that while the University seems to be embracing the HWE, it is failing to provide academics with data to enable them to virtually attend meetings while working remotely. One participant highlighted that this is very prevalent in junior academics who in most cases will not join meetings due to a lack of data as they need to provide themselves with data while working from home.

*“The university does not provide or compensate you with data when you are working from home but expect you to attend some of these meetings and send students feedback…resources do not commensurate to what you are supposed to be doing”* (RP9, male, 36 years, senior lecturer, Vanderbijlpark campus).

*“… those who are juniors will try not to be online most often because data comes from their own pockets, and you will not know what is happening to them… supporting them with data is important, one will be able to reach them anytime”* (RP12, male, 44 years, senior lecturer, Potchefstroom campus).

Participants also noted that the University only provides them with a laptop yet working remotely in the hybrid requires one to have a printer as well, among other resources that the University is not providing.

*“Academics need to be funded to get other equipment like printers while working from home as these are also important… not getting them makes me feel like am not important to the university”* (RP2, female, 55 years, lecturer, Vanderbijlpark campus).

*“… I will feel more valuable to the organisation if I was to get a printer at home and a network system which is mobile that I can use anywhere without having to pay it myself”* (RP10, female, 39 years, lecturer, Vanderbijlpark campus).

Three participants also noted that time as a resource is not available to academics because of the HWE. The participants noted that the University can offer various equipment to get the work done in the HWE but the resource that is called time has been very scarce since the inception of the HWE and this harms the sense of belonging.

*“Time is another resource; you can have all the resources and but do not have time you will feel overwhelmed and overworked and you feel the university is more concerned about outputs than me as an individual”* (RP1, female, 52 years, lecturer, Potchefstroom campus).

*“… yes, workshops are happening almost every day but as an academic there is no time to attend all these”* (RP11, male, 58 years, associate professor, Mahikeng campus).

*“… academics have a lot to do yet there is no time….”* (RP5, female, 40 years, lecturer, Potchefstroom campus).

Findings of the study indicate that academics lacked resources such as data and other office supplies like printers that enabled them to perform optimally while working remotely. Time as a resource was also lacking among academics in the HWE which impacted negatively on opportunities to engage in other activities that enhanced their social well-being at the workplace. Ahmad et al. (2020) note that time is a critical resource that is reduced due to work pressures while working in the HWE. Failure to provide resources makes employees feel isolated and the provision of resources enhances work flexibility in the HWE which creates autonomy thereby enhancing belonging in the organisation (Ahmad et al., 2020). According to the JD-R model, the provision of resources at the workplace mitigates pressure that is exerted on employees by job demands. Therefore, the lack of resources in the HWE is a challenge that impacts the well-being of academics. The findings of the study are in line with those of Wigert and White (2022) who reported that 35% of their research participants indicated that they had less access to resources and equipment because of the HWE.

### Work life imbalance

Participants noted that the HWE has impacted their work-life balance because of blurred boundaries that result when one works in the HWE. According to some research participants, embeddedness in the University is being compromised by the work-life imbalance.

*“… The so-called flexibility in the HWE comes with a price, at times you drive in a meeting, … last night I was in a meeting with my supervisor which ended at 10 pm”* (RP9, male, 36 years, senior lecturer, Potchefstroom campus).

*“If I want to write an article, I have to do it during family time which means I have to take my personal family time and there is no sense of belonging in a case like that”* (RP1, female, 52 years, lecturer, Potchefstroom campus).

The findings of the study are contradictory to Laß and Wooden (2023) who found that the HWE enhances work-life balance. The contradiction may be because their study was longitudinal while this study was cross-sectional as well as the different industries covered by the studies. The academic industry is characterized by a high workload. However, the findings are in line with Ahmad et al. (2020) study among teleworkers in the IT industry which found that the HWE blurred work and personal life boundaries. Work-life balance is an important job resource critical in the HWE (Babapour Chafi et al., 2022). The findings are in line with the JD-R model and work-life balance is a critical job resource that buffers work demands.

### Onboarding challenges

Some participants noted that social isolation challenges and a compromised sense of belonging in the HWE are prevalent in onboarding of new employees. Two participants noted that they were hired at the inception of the HWE, and they do not physically know most of their work colleagues except their email addresses and cannot put a face to the email address.

*“I think it’s horrible for people who are new. We do not even know who is new in terms of physically meeting them we only know of them if ever an email is sent that there is someone new and it’s difficult to connect with them”* (RP4, female, 37 years, lecturer, Vanderbijlpark campus).

*“I joined the university in 2020 during COVID-19 for a long period of time I never knew any colleagues, there was zero work interactions, and I was not able to connect with my work colleagues”* (RP8, male, 47 years, lecturer, Vanderbijlpark campus).

According to Fayard et al. (2021), new employees are hesitant to send emails to colleagues they have never met. This impacts their opportunities to connect even virtually which is detrimental to their sense of belonging. Study findings are congruent with findings by Babapour Chafi et al. (2022). Research participants in the study highlighted that trust and relationships were difficult to build with new employees due to limited connections because of the HWE. Fayard et al.’s (2021) study cites a lack of information on the organisation and its systems due to online boarding in the HWE. Lack of such information affects new employees’ sense of belonging. Information is an important job resource that mitigates job demands and hence the findings are in line with the JD-R model.

### Lack of an inclusive work environment

Research participants indicated that their sense of belonging to the University is compromised by management who do not include academics when making decisions and pretend to be consulting when in fact decisions would have been made. This non-consultative decision-making made academics feel less valued in the HWE and harmed their sense of belonging. One participant noted:

*“… with the HWE management is taking us for granted now, they no longer consult us and when they do they would have made the decision already….”* (RP1, female, 52 years, lecturer, Potchefstroom campus).

Waller (2020) argues that involvement in decision-making enhances the perception of being valued among employees.

## Relationships

Work relationships, perceived trust and social support are critical elements of employee social well-being (Tabor-Błażewicz, 2023). The ability to communicate, develop meaningful work relationships and have a strong support network at the workplace enhance the social well-being of employees (Tabor-Błażewicz, 2023). According to Mikkola and Nykänen (2019) workplace relationships are an important resource. Keppler and Leonardi (2023) highlight that despite the importance of work relationships in the HWE, hybrid workers know a few of their work colleagues and have limited positive work relationships. Three sub-themes emerged: lack of trust, lack of social support, and delayed feedback.

### Lack of trust

Participants noted that the HWE reduces trust among work colleagues, and this harms relationships at the workplace. One participant highlighted that while communication takes place using virtual platforms, it is difficult to open up to your supervisors during conversations as one cannot tell who else will be listening in on their meeting.

*“Trust has been affected… I may talk to someone online and not know who else is in the room and I may not open up because I do not know who is in the background”* (RP1, female, 52 years, lecturer, Potchefstroom campus).

*“Social distance resulting from this hybrid makes it difficult to trust each other, when you do not see people often it reduces your trust on them”* (RP6, female, 33 years, lecturer, Vanderbijlpark campus).

Ofosu (2023) highlights that trust is imperative in the HWE as it helps to maintain healthy work relationships, among other benefits. Similarly, Mishra and Bharti (2023) concur that trust is a critical commodity in the HWE. Waller (2020) posits that leadership is important in creating psychological safety which impact on trust among work colleagues.

### Lack of social support

Some participants noted that the lack of social support that is inherent in the HWE has affected positive work relationships. Participants noted that getting social support from work colleagues helps to build social relationships and sustain those that already exist. However, the HWE made it difficult for academics to offer or request social support from colleagues.

*“Everyone now minds their own business, and it has diminished relationships among colleagues”* (RP9, male, 36 years, senior lecturer, Vanderbijlpark campus).

*“Previously you could just go to a colleague’s office and seek help… with hybrid you may not find people in offices”* (RP6, female, 33 years, lecturer, Vanderbijlpark campus).

Another participant noted that with the HWE it is difficult to offer support to colleagues because there is limited information on colleagues. The participant noted that when one is not in the office one can just assume someone is working from home, yet someone could be in the hospital or bereaving, which makes it difficult to offer them support, which is important in building relationships with colleagues.

*“At times you do not even know what your colleague is going through personally…you fail to support them and that affects building good relationships”* (RP8, male, 47 years, lecturer, Vanderbijlpark campus).

Wu et al. (2023) highlight that the HWE is characterized by limited social support. According to Ahmad et al. (2020), social support provides emotional and instrumental support which when given reduces loneliness and connects employees. Connections afford employees to build relationships. Findings are in line with the JD-R model and the JDCS model. Social support is a critical resource that can be used to mitigate job demands.

### Delayed feedback

Some participants felt that the HWE delays feedback among work colleagues. Feedback that could have been spontaneous by popping into someone’s office now takes time. One participant noted that the provision of timely feedback impacts relationships in organisations.

*“Before this HWE, one could just meet with your colleague next door and get a solution quickly… turnaround time to get feedback has been affected”* (RP8, male, 47 years, lecturer, Vanderbijlpark campus).

*“We no longer have those informal meetings where you meet a colleague for a quick inquiry to have immediate feedback”* (RP22, 39 years, senior lecturer, Potchefstroom campus).

Findings indicate that the HWE is characterized by delayed feedback. The delayed feedback results from reduced interactions among academics in the HWE. Wigert and White (2022) state that impaired feedback is one of the common challenges that hybrid workers experience. Participants of a study by Babapour Chafi et al. (2022) cited that they received limited feedback when they were working remotely. A study by Fayard et al. (2021) found that the HWE was detrimental in providing timely feedback to new employees. The feedback that could be given in 10 s was delayed due to the back and forth of several emails. Eva et al. (2019) highlight that feedback is a critical job resource. Taking feedback as a resource that buffers job demands one can assert that the findings are in line with the JD-R model and the JDCS model.

## Discussion of results

The aim of the study was to explore and understand social employee well-being challenges that academics face due to the HWE. The study’s results revealed that while HEIs have widely adopted hybrid work models, these arrangements have compromised the social well-being of academics. The study showed that differences in work location have adversely affected academics’ sense of belonging, work relationships, and social connections. These have been harmful to academics’ overall social well-being at the workplace.

The findings of the study highlighted that academics value social interactions or connections, and these have been negatively altered by the HWE. Academics experience some social isolation at the workplace due to the hybrid work settings. Jaworski et al. (2022) argue that while social connections are critical to employee well-being, their form and medium have been altered by the HWE. The autonomy to choose where academics work has caused social isolation among academics – it has reduced face-to-face interactions and caused academics to feel alienated thereby negatively impacting social interactions or connections among academics. The study’s findings indicated that the HWE has reduced academics’ opportunities to interact. According to Jaworski et al. (2022), spontaneous interactions are not easy in the HWE and their negative impact on building work relationships cannot be undermined. Similarly, Zajac et al. (2022) agree that the HWE creates a distance among co-workers making social connections difficult. This calls for organisational leaders to ensure that intentional anchored physical meetings are incorporated to provide employees with opportunities to interact. Where possible virtual interactions should be incorporated to mitigate reduced interactions resulting from the HWE.

Reduced informal communication due to the HWE impacts academics’ opportunities to connect. Findings of the study are congruent with Connor et al. (2022) study which concluded that the HWE reduced spontaneous and informal communication among work colleagues in Sweden and Ireland. This affects their social well-being as informal communication is associated with job satisfaction and organisational commitment (Zajac et al., 2022). Job satisfaction and organisational commitment are outputs of a sense of belonging among employees. Networking among academics has also been affected by the HWE and has magnified social isolation risks among academics. Jaworski et al. (2022) contend that the HWE reduced networking opportunities for employees. The flexibility and autonomy that come with the HWE are detrimental to the social well-being of academics, especially when considering that social connections are a dimension of social employee well-being. This shows that the nature or characteristics of the HWE and not organisational factors heighten the risk of reduced social connections or interactions among employees.

Findings also indicated that the sense of belonging of academics in the HWE is negatively impacted by lack of resources, work-life imbalance, onboarding difficulties as well as a non-inclusive work environment. Academics felt that the university did not provide adequate resources to function optimally and time as a resource was insufficient for academics in the hybrid work environment. Findings of the study are in line with a Microsoft (2021) study which found that 42% of employees working in the HWE lack necessary office supplies while 10% did not have a good internet connection. According to Kokt and Chipunza (2022), work resources are critical in the hybrid work environment, and these are scarce, especially in the African context. One can contend that academics have been exposed to a lack of resources because of the context that they are working from and the nortion that the higher education industry was under-resourced even before the HWE. Naidoo-Chetty and du Plessis (2021) note that the industry is under-resourced. Hence a lack of resources in the HWE is a challenge for academics working in a hybrid work environment. The lack of resources as a challenge in the HWE has been magnified by the nature of the industry as well as the context. Therefore, social well-being challenges can be caused by external factors. Hence when exploring these challenges, it is salient to explore the organisational as well as external factors. This indicates that organisational leaders need to provide employees with job resources that buffer effects of the job demands prevalent in the HWE.

The flexibility that comes with the HWE impacted negatively on the work-life balance of academics. Findings of the study indicated that the HWE impacted negatively on work-life balance among academics. While the findings of the study contradict Laß and Wooden (2023), findings they are in line with Ahmad et al. (2020) study that noted work life imbalance among I.T professionals as a result of the HWE. OECD (2023) highlights that work-life balance is critical for well-being. However, this critical element of employee well-being has been compromised by the HWE. Rupčić (2024) notes that one of the limitations of the HWE is mixing professional and private life. While some authors claim that the HWE enhances work-life balance (Zwanka and Buff, 2020; Gorjifard and Crawford, 2021; Budhkar and Salve, 2023), it is important to note that the academic profession has been cited as a profession that fails to separate life and work responsibilities (Diego-Medrano and Salazar, 2021). Hence, the nature of the industry or the quality of the job contributes to social well-being challenges that academics face in the hybrid work environment. This shows the need to incorporate various factors contributing to social challenges in the HWE. Organisational leaders need to incorporate and explore various factors when addressing social well-being challenges in the HWE as factors such as the nature of industry which intensify the magnitude of challenges faced.

The findings of the study also highlighted that academics’ sense of belonging is affected by onboarding challenges in the HWE which negatively impacts their sense of belonging. Klinghoffer et al. (2024) indicate that onboarding is one of the processes that have been affected by the HWE. With employees working in different locations, new employees cannot build connections with their work colleagues which leads to social isolation and negatively affects their sense of belonging. New employees experiencing onboarding challenges highlights the need to have primary interventions in managing social well-being; this helps HEIs to be proactive in dealing with social well-being challenges. This also shows that social well-being challenges are more intensified on new employees as they have to deal with onboarding difficulties on top of other challenges that all academics may be facing. It also reveals the importance of social capital in the HWE which new employees will not be having when they start at a new organisation. This calls for organisational leaders and policy makers to ensure that when addressing social well-being challenges in the HWE it is important to complement secondary interventions with primary interventions. Traditional approaches to onboarding need to be re-evaluated. Onboarding should compensate and complement the HWE, personalized onboarding will create a strong sense of connection among employees while enhancing their sense of belonging.

The HWE has not spared work relationships. The findings of the study indicated that academics’ work relationships have been negatively affected. Work relationships have been weakened by the lack of trust, reduced social support and delayed feedback that is prevalent in the HWE. Findings highlighted that trust has been reduced between employees and management in the new work environment as the tools used for communication make it difficult for employees to express themselves. Findings of the study are in line with Babapour Chafi et al. (2022) study among Swedish employees. The participants noted that reduced face-to-face interactions and limited feedback made it difficult for employees to build trust and work relationships. In other words, this lack of trust has affected academics’ psychological safety. According to Cazan (2023), psychological safety is a bedrock for positive work relations between organisational leaders and employees. Horizontal and vertical trust among employees enhances social integration via psychological safety (Hennicks et al., 2024). Teng-Calleja et al. (2024) argue that trust is a challenge in the HWE. According to Hennicks et al. (2024), lack of trust negatively impacts social equilibrium which harms work relationships. Therefore, lack of trust in the HWE impacts negatively on the social well-being of academics as it dents positive and meaningful work relationships. One can also conclude that trust is a critical issue in the success of the HWE and the social well-being thereof. The salience of trust requires organisational leaders to invest more in activities and events that enhance frequent in person interactions which consequently builds trust among employees in the HWE.

Delayed feedback was cited as one of the challenges that academics face in the hybrid work environment, which negatively impacted work relationships and the social well-being of academics in the HWE. This is in line with Teng-Calleja et al. (2024) study among middle managers in the Philippines. Research participants highlighted that the HWE reduced opportunities to receive immediate feedback. Provision of feedback enhances sense of belonging and mitigates job demands thereby enhancing well-being (Waller, 2020; Eva et al., 2019). Teng-Calleja et al. (2024) note that delayed feedback in the HWE emanates from unresponsive work colleagues as a result of limited communication and collaboration. This shows that some of the social challenges that academics face result from the personal traits of individuals and hence the need to incorporate personality traits when exploring the nature and magnitude of social well-being challenges in the HWE.

In summary, the findings of the study highlight that challenges faced by academics in the HWE have a ripple effect on the three dimensions of social well-being. Social isolation negatively impacts social connections – an important element of social well-being – which in turn negatively impacts the sense of belonging in academics and the maintenance of positive work relationships. The findings also depict that though all academics experience social well-being challenges, social well-being challenges of new academics are heightened by onboarding challenges which harm their social connections, sense of belonging and work relationships. Last but not least, personality traits and other macro factors contribute to challenges that academics are facing due to the HWE. Extroverts felt the HWE inhibited them from connecting with work colleagues while introverts did not experience any isolation as a result of the HWE. Additionally, the context of the study, Africa, which is characterized by a lack of resources contributed to the magnitude of social well-being challenges that employees face in the HWE.

## Limitations and recommendations

The participants for the study were solely selected from one public higher education institution in South Africa. Qualitative research methods with a limited sample size were utilized, affecting the study’s generalisability; the findings can still apply to other research contexts. The study’s use of the purposive sampling method posed a risk of selection bias, potentially leading to the chosen population not fully representing the target population. The study was cross-sectional and focused on academics in the HWE at a particular point in time, however, this did not impact the findings. It is worth noting that conducting a longitudinal study would have provided a more comprehensive understanding of the subject matter.

The current study examined the social well-being of academics in South Africa, specifically at public institutions. Future research could investigate academics at private institutions, as social well-being issues may differ between the private and public sectors. This shows the need to conduct comparative studies to gain a holistic understanding of social well-being challenges that academics face in the HWE. Future research can consider adopting a quantitative research approach to enhance generalizability of research findings. There is limited research on the support staff at HEIS, further research could explore the social employee well-being of support staff at HEIS, given their crucial role in the sustainability and competitiveness of these institutions. Future research could also examine factors that are personal to academics, such as personal resources and personality traits in the HWE. This also calls for further research on how social well-being challenges may be distinct for each generation. Conducting a longitudinal study is also recommended as it will provide a more comprehensive understanding of the subject matter.

## Conclusion

While the HWE is being embraced across the globe, it has several drawbacks. Most of these drawbacks negatively impact on employee’s opportunities for social connections, creating and sustaining positive work relationships as well as enhancing sense of belonging. This indicates that the HWE impacts negatively on the social well-being of academics. The social well-being challenges faced by academics in the HWE were intensified by the nature of the industry, review of literature highlighted that academics’ social employee well-being was in a compromised state and the HWE intensified the compromised social employee well-being of academics. The context in which organisations operate in also impacted on the challenges that employees face while working in the HWE. Lastly, some traditional approaches to talent management also perpetuate social well-being challenges that employees face while working in the HWE.

## Data Availability

The raw data supporting the conclusions of this article will be made available by the authors, without undue reservation.
